# Beta-2 adrenergic receptor gene polymorphisms Gln27Glu, Arg16Gly in patients with heart failure

**DOI:** 10.1186/1471-2261-9-50

**Published:** 2009-11-03

**Authors:** Alfredo José Mansur, Rosana Seleri Fontes, Regina Airoldi Canzi, Raphael Nishimura, Airlane Pereira Alencar, Antonio Carlos Pedroso de Lima, José Eduardo Krieger, Alexandre Costa Pereira

**Affiliations:** 1 Heart Institute (InCor), University of São Paulo Medical School, São Paulo, Brazil; 2 Institute of Mathematics and Statistics, University of São Paulo, São Paulo, Brazil

## Abstract

**Background -:**

Beta-2 adrenergic receptor gene polymorphisms Gln27Glu, Arg16Gly and Thr164Ile were suggested to have an effect in heart failure. We evaluated these polymorphisms relative to clinical characteristics and prognosis of alarge cohort of patients with heart failure of different etiologies.

**Methods -:**

We studied 501 patients with heart failure of different etiologies. Mean age was 58 years (standard deviation 14.4 years), 298 (60%) were men. Polymorphisms were identified by polymerase chain reaction-restriction fragment length polymorphism.

**Results -:**

During the mean follow-up of 12.6 months (standard deviation 10.3 months), 188 (38%) patients died. Distribution of genotypes of polymorphism Arg16Gly was different relative to body mass index (χ^2 ^= 9.797;p = 0.04). Overall the probability of survival was not significantly predicted by genotypes of Gln27Glu, Arg16Gly, or Thr164Ile. Allele and haplotype analysis also did not disclose any significant difference regarding mortality. Exploratory analysis through classification trees pointed towards a potential association between the Gln27Glu polymorphism and mortality in older individuals.

**Conclusion -:**

In this study sample, we were not able to demonstrate an overall influence of polymorphisms Gln27Glu and Arg16Gly of beta-2 receptor gene on prognosis. Nevertheless, Gln27Glu polymorphism may have a potential predictive value in older individuals.

## Background

Genetic polymorphisms have been studied with the hypotheses of influencing presentation, course and prognosis of patients with heart failure [1]. Beta-2 adrenergic receptor gene polymorphisms Gln27Glu, Arg16Gly, and Thr164Ile were suggested to have a significant effect in patients with heart failure. In a study of 259 patients with ischemic or idiopathic dilated cardiomyopathy, prognosis was less favorable in patients harboring the allele Ile164 relative to patients harboring the allele Thr164. In patients harboring alleles Gly16 and Glu27, previoulsy associated with increased down regulation of beta-2 adrenergic receptors, a statistically significant difference relative to prognosis has not been demonstrated. However, a trend to a prognostic influence of allele Gly16 was suggested by an apparent and non-significant lower survival rate in patients harboring allele Gly16 [2]. The polymorphisms Thr164Ile, Arg16Gly and the haplotypes with alleles Gly16 and Gln27 were associated with reduced exercise tolerance in other study that enrolled 232 patients with idiopathic dilated cardiomyopathy or ischemic cardiomyopathy [3]. These findings substantiate the hypotheses that polymorphisms Arg16Gly, Gln27Glu and Thr164Ile of beta-2 receptor gene might be functional and might be associated with differences in the survival rate of patients with heart failure. Therefore, due to sparse and sometimes conflicting results, this hypothesis deserves further testing in different populations.

We performed this study to evaluate the polymorphisms Gln27Glu, Arg16Gly, and Thr164Ile of the beta-2 receptor gene relative to demographic and clinical variables, as well as, survival rate in a series of patients with heart failure of different etiologies.

## Methods

### Study population

501 patients with heart failure in functional class III or IV of the New York Heart Association were studied. Patients aged 58 (SD 14.4) (minimum 18; maximum 93; median 59) years, 298 (60%) were men and 203 women. The patients were included in the study from August, 2002 to March, 2004.

The diagnosis of heart failure was made according to previously published criteria [4]. The classification of the etiologies of heart failure followed previous recommendations [5-7]. As such, the diagnosis of chronic heart failure was made through both clinical and imaging procedures when necessary. Ischemic cardiomyopathy diagnosis this was made when a clear history of previous myocardial infarction and no other probable cause of heart dysfunction was present or, alternatively, through coronary angiography. All patients with the final diagnosis of idiopathic dilated cardiomyopathy were studied through coronary angiography to exclude the diagnosis of ischemic cardiomyopathy.

### Clinical characteristics of the patients

367 (73%) patients were white, 121 (24%) were mullattoes, and 13 (3%) were from other ethnicities.

Hypertensive cardiomyopathy was diagnosed in 144 (29%) patients, ischemic cardiomyopathy in 143 (29%), cardiomyopathy due to valve disease in 76 (15%), Chagas'heart disease in 60 (12%). Other etiologies were diagnosed in 28 (6%) patients. Heart failure was ascribed to idiopathic dilated cardiomyopathy in 50 (10%) patients. One-hundred and twenty nine (26%) patients were diabetic (type II).

The distribution of body mass index (evaluated in 362 patients) was: < 18.5 kg/m^2 ^in 26 (7%) patients, 18.5-25 kg/m^2 ^in 160 (44%), 25-30 kg/m^2 ^in 112 (31%) and > 30 kg/m^2 ^in 64 (18%).

Sinus cardiac rhythm was diagnosed in 304 (61%) patients, atrial flutter or fibrillation in 123 (25%) and was not retrieved in 74 (15%).

Drug regimens used in therapy at enrollment (baseline of follow-up) included diuretics (mainly furosemide) in 356 (71%) patients, angiotensin converting enzyme inhibitors in 330 (66%), digoxin in 264 (52%), antiplatelet agents 113 (22%), beta-blockers (mainly carvedilol) in 102 (20%), warfarin in 96 (19%), spironolactone in 77 (15%), nitrates in 75 (15%), other vasodilators in 49 (10%), amiodarone in 40 (8%). After enrollment and during follow-up angiotensin converting enzyme inhibitors, angiotensin II receptor blockers, beta-blockers and spironolactone were titrated until maximum dose or dose tolerated by patients was achieved.

Other clinical and laboratory characteristics of the patients in this study sample are shown in Table 1.

**Table 1:** Clinical and laboratory characteristics of the study sample

**Variable**	**n**	**Mean**	**Standard deviation**
Age (years)	501	58	14.4
Body weight (kg)	414	67.6	15.7
Height (m)	371	1.63	0.09
Body mass index (kg/m^2^)	362	25.6	5.6
Heart rate, bpm	431	79.6	13
Blood pressure (mm Hg)			
Diastolic	450	76	18.4
Systolic	450	121.9	30.9
Echocardiography			
Interventricular septum (cm)	435	0.97	0.23
Left ventricle			
Posterior wall (cm)	435	0.95	0.2
Diastolic diameter (cm)	444	6.2	1.15
Systolic diameter (cm)	338	4.97	1.42
Ejection fraction (%)	397	45	19
Interventricular septum (cm)	435	0.97	0.23
Aorta (cm)	425	3.26	0.59
Left atrium (cm)	438	4.68	0.9
Right ventricle (cm)	192	2.73	0.87
Serum sodium (mEq/L)	481	136.7	4.5
Hemoglobin (g/dL)	479	13.07	2.1
Serum cholesterol (mg/dL)	400	190.1	51.6
Triglycerides (mg/dL)	398	123.1	66.7
HDL-cholesterol (mg/dL)	393	45.1	15.4
LDL-cholesterol (mg/dL)	394	120.1	42
VLDL-cholesterol (mg/dL)	389	24.8	13.6
Serum creatinine (mg/dL)	487	1.3	0.74

### Genotype determination

Genomic DNA was extracted from leukocytes in samples from whole blood, following standard techniques. The three studied polymorphisms were detected by polymerase chain reaction-restriction fragment length polymorphism, as previously described [8].

Quality control for these assays was assessed by randomly selecting 50 samples to be re-genotyped by two independent technicians.

### Studied variables

Polymorphisms Gln27Glu, Arg16Gly, and Thr164Ile of beta-2 adrenergic receptor gene were studied relative to sex, ethnicity, age, duration of symptoms, etiology of heart failure, diabetes mellitus, history of arterial hypertension, smoking, body mass index (kg/m^2^), heart rate, blood pressure, cardiac rhythm, cardiac dimensions on echocardiography, left ventricle ejection fraction, serum sodium, hemoglobin, serum cholesterol and triglycerides, HDL-cholesterol, LDL-cholesterol, VLDL-cholesterol, serum glucose, creatinine, drug therapy, and outcome.

### Follow-up

The follow-up was assessed in the last outpatient medical visit or by telephone contact. In addition, the mortality database of São Paulo City Authority was also scrutinized to discover patient deaths (ProAim--Programa de Aprimoramento de Informações de Mortalidade do Municipio de São Paulo). Last follow-up was evaluated in April, 2006. Main end-point studied was overall mortality.

### Statistical analysis

After descriptive statistics to evaluate the sample profile, the distribution of polymorphisms was tested for Hardy Weinberg equilibrium. The distribution of genotypes was compared to population samples reported in previous studies [9]. Comparison of proportions was used to test the distribution of the studied genotypes relative to other demographic and clinical variables. Haplotype determination was conducted using the Haploview software (version 4.1).

The probability of survival was evaluated by the Kaplan Meier method relative to demographic and clinical variables and relative to genotypes and alleles of studied polymorphism. Comparisons were made with the log-rank test. Death (overall) was considered an event.

A Cox proportional hazard regression model was adjusted for assessing the independent variables associated with prognosis; variables were selected through stepwise as well as a manual procedure (in the order they appear in Table 2).

**Table 2:** Mortality relative to studied variables including information derived from exploratory analysis (Cox proportional hazards model)

**Variables**	**p**	**Hazard ratio**	**95% Confidence interval**
Serum sodium	< 0.001	0.94	(0.91; 0.96)
Serum creatinine	< 0.001	1.47	(1.27; 1.69)
Etiology	0.09	0.91	(0.81; 1.02)
Ethnicity	0.73	0.97	(0.80; 1.17)
Gln27Glu ^#^	0.01	5.48	(1.50; 20.08)
Age	< 0.001	1.03	(1.02; 1.05)
Age * Gln27Glu ^#^	0.004	0.97	(0.95; 0.99)
Baseline beta-blocker use	0.001	2.30	(1.43; 3.71)

^# ^dominant model (GlnGln genotype reference versus GlnGlu + GluGlu genotypes)

Serum sodium, serum creatinine and age included as continuous variables

Etiology (1. Chagasic, 2 Idiopathic, 3 Hypertensive, 4 Isquemic, 5 Heart failure due to valve disease)

Classification trees were used for exploratory analysis of potential interactions in regarding to mortality. Used growing method was CHAID. Significance level for splitting nodes was 0,05 and for merging categories was 0,05. Model estimation was performed with the maximum number of iterations of 100, and Bonferroni method was used to adjust for multiple comparisons.

Statistical analyses were performed with R software [9] or SPSS statistical package 13.0. A *p *value < 0.05 was considered significant.

### Ethics

The study protocol was approved by the Ethics Committee for Medical Research of the Hospital das Clinicas Universidade de São Paulo. Informed consent was obtained and signed by all participants.

## Results

### Distribution of alleles and genotypes

The distribution of allele frequencies, genotypes, and haplotypes are shown in Table 3. For polymorphism Thr164Ile, 97% of the samples were homozygous for the Thr164Thr genotype and 3% heterozygous for Thr164Ile; this polymorphism was excluded from further analysis by the low frequency of heterozygous genotypes. The frequencies of polymorphisms Gln27Glu and Arg16Gly were in accordance with Hardy Weinberg equilibrium.

**Table 3:** Allelic and genotypic frequencies of polymorphisms Gln27Glu, Arg16Gly, and Thr164Ile in the study sample of patients with heart failure

**Allelic frequencies**	**Patients** **N (%)**		
Arg	421 (42)		
Gly	583 (58)		
Gln	702 (71)		
Glu	290 (29)		
Thr	983 (99)		
Ile	9 (1)		
			
**Genotypic frequencies**	**N (%)**	**N (%)**	**N (%)**
Arg16Gly	Arg16Arg 98 (20)	Arg16Gly 225 (45)	Gly16Gly 179 (35)
Gln27Glu	Gln27Gln 253 (51)	Gln27Glu 196 (39)	Glu27Glu 47 (9)
Thr164Ile	Thr164Thr 487 (98)	Thr164Ile 9 (2)	Ile164Ile - (-)
			
Haplotypic frequencies	(%)		
Arg16/Gln27	40		
Arg16/Glu27	2		
Gly16/Gln27	30		
Gly16/Glu27	28		

* samples lost due to technical difficulties

The difference in the distribution of the genotypes of polymorphism Gln27Glu was statistically significant relative to ethnicity (χ^2 ^= 9.965; p = 0.04); the difference was not significant for genotypes of polymorphism Arg16Gly. The distribution of genotypes of polymorphism Arg16Gly was statistically significant relative to body mass index analyzed as an ordinal variable (χ^2 ^= 9.797; p = 0.04, being genotype Arg/Arg associated with a higher frequency of a BMI above 25), but not when it was analyzed as a continuous variable (BMI mean values (SD): Arg/Arg 26.32 (5.75), Arg/Gly 25.61 (5.44), Gly/Gly 25.14 (5.13); p = 0.34); no significant difference was observed for genotypes of polymorphism Gln27Glu.

The difference in the distribution of genotypes of both polymorphisms Arg16Gly and Gln27Glu was not statistically significant relative to diabetes mellitus, history of arterial hypertension, smoking, cardiac rhythm, etiology of heart failure, and mortality. Detailed information regarding frequency, means and standard deviations for demographic and biochemical variables per genotypic groups are available as Supplementary information (see Additional file 1).

### Outcome

188 (38%) of 501 patients died during the mean follow-up of 12.6 (standard deviation 10.3; median 11) months.

The probability of survival revealed a statistically significant differencerelative to the etiologies of heart failure (log-rank, p = 0.02) (individuals with the diagnosis of Idiopathic Dilated Cardiomyopathy having the worst prognosis). The probability of survival did not reveal a statistically significant difference relative to the 3 genotypes of polymorphism Gln27Glu (Gln27Gln, Gln27Glu, Glu27Glu) and relative to the three genotypes of polymorphism Arg16Gly (Arg16Arg, Arg16Gly, Gly16Gly).

A Cox proportional hazards model constructed for the entire population revealed that the variables significantly associated with prognosis were serum sodium (higher or under 136 mEq/l), serum creatinine, and etiology of heart failure (data not shown). These variables were used for adjustment in models derived from the exploratory analysis.

**Figure 1:**
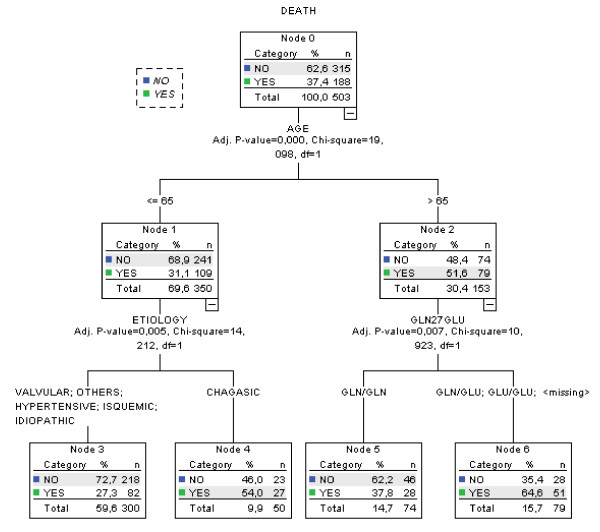
**Classification Tree derived from exploratory analysis**.

Next, we have performed exploratory analysis through classification trees with the aim of identifying a potential subgroup of heart failure patients where B2 adrenergic genotype might be of predictive value. For this we have used in model's construction the following variables: genotype for the Arg16Gly polymorphism, genotype for the Gln27Glu polymorphism, ethnicity, age, sex, and HF etiology. Interestingly, the best classification model selected (Figure 1) incorporated information from the Gln27Glu polymorphism. The genotypic information derived from this marker was able to sub-classify individuals older than 65 years into a mortality high-risk group (65% chance of dying) and an intermediate-risk group (38% chance of dying).

**Figure 2:**
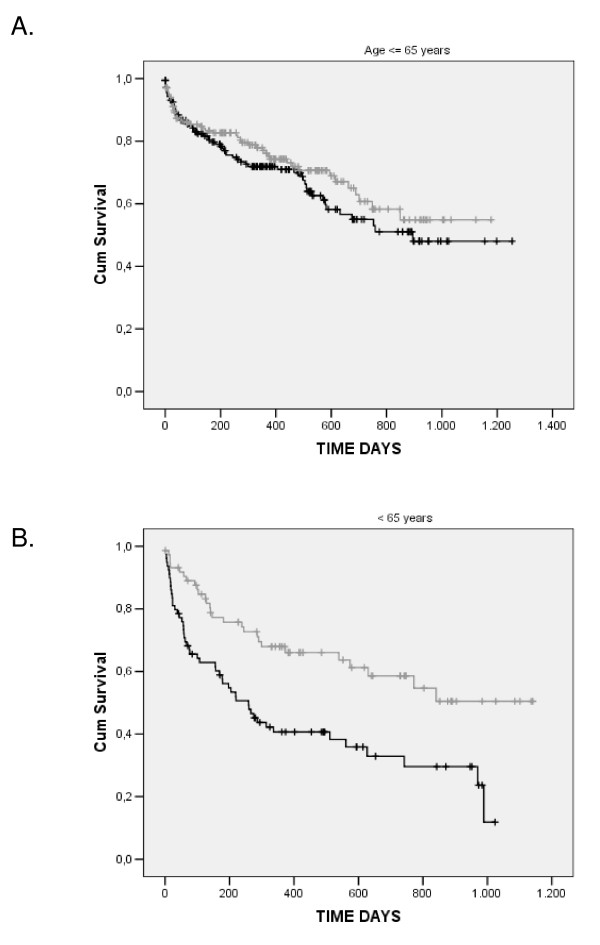
**Kaplan-Meir survival curves for the Gln27Glu polymorphism per age group**. Gray lines: GlnGln genotype; black lines: GlnGlu + GluGlu genotypes.

We have tested the use of this information through modeling time to event (death) in the entire population in a new Cox regression model, but adding both Gln27Glu information and the interaction term Gln27Glu*age in the model. In this new model (using data from 471 cases and 185 events), not only the interaction term was significantly associated with mortality, but the marginal term for the Gln27Glu polymorphism was also associated with heart failure mortality in our population. Both terms remained significantly associated with mortality even after adjustment for age, serum sodium, serum creatinine, ethnicity, baseline beta-blocker use, and heart failure etiology (Table 2). In fact, while a significant difference in relation to overall mortality was not observed for Gln27Glu genotypes in heart failure individuals younger than 65 years of age, a clear difference was observed when only individuals older than 65 years were analyzed (a subgroup comprising 30% of the studied sample) (Figure 2). In addition, a tendency towards increased mortality of Glu allele carriers was present in older individuals with both systolic and diastolic types of heart failure included in our cohort.

Taking into account the linkage disequilibrium between Arg16Gly and Gln27Glu (in our population of 93), our data point to an effect derived specifically form the Glu27 allele. First, allele analysis in older individuals were only able to disclose a significant association for the Glu27 allele (p = 0,002 for mortality), but not for the Arg16 or Gly16 allele (p = 0,33). Second, haplotype analysis showed a higher frequency of the Gly16/Glu27 haplotype in individuals who died (p = 0,001), but a lower frequency of the Gly16/Gln27 in individuals that died (p = 0,03). No difference was observed for the Arg16/Gln27 between patients who died and patients that did not, and the Arg16/Glu27 was, as expected, very rare in these individuals. These data suggest that, in fact, the effect was only present for the Gln27Glu polymorphism, and not derived indirectly from the strong LD pattern with the Arg16Gly polymorphism.

## Discussion

The different genotypes of polymorphisms Gln27Glu and Arg16Gly were present in the study sample in a distribution in accordance with previous reports including a Brazilian population sample [9]. Most genotypes of polymorphism Thr164Ile were homozygous for Thr164Thr; no patient homozygous for Ile164Ile was identified. Thr164Ile was identified only in 9 patients (2%), a lower frequency than observed in a previous experiment [3]. The number was considered small to the point of hindering appropriate statistical analysis and further analysis of this polymorphism relative to survival rate was not performed.

We observed a significant difference in the distribution of the polymorphism Arg16Gly relative to body mass index in this study sample of patients with heart failure. In a previous study of a general population sample, Arg16Gly polymorphism interacted significantly with body mass index to determine systolic blood pressure. Further, the presence of Arg16 allele was associated with 1.49-fold increase in the risk of obesity in multiple logistic regression model [9]. Thus, the relationship between the presence of Arg16 and body mass index may be operative also in patients with heart failure. Nevertheless, we have not observed a significant effect of this genetic marker in relation to mortality.

We were not able to demonstrate a significant association between survival rate for the overall sample and the different genotypes, alleles or haplotypes of polymorphisms Gln27Glu and Arg16Gly in this study sample at the significance level of p < 0.05. The significant variables associated with mortality - low body mass index [10], etiology of heart failure [11], serum sodium [12], serum creatinine [13] are known from previous experiences. Interestingly, we have observed a potential interaction between age and Gln27Glu polymorphism in modulating mortality incidence of the studied patients. In this scenario, the Gln27Glu polymorphism is predictive of a higher mortality only in individuals older than 65 years of age. Allele and haplotype analysis suggest that the main predictor of this effect was the Gln27Glu polymorphism and not, indirectly, the Arg16Gly polymorphism.

Similar to Ligget et al. [2] and to De Groote et al. [14] we did not find a significant association between the Arg16Gly and Gln27Glu polymorphism and survival when analyzing the entire population. Different from the described by De Groote et al., however, we observed a tendency of a lower survival rate in the Gly16Glu27 haplotype and not the Gly16Gln27 haplotype [14]. Nevertheless, we are not the first group to describe this particular association. Forleo et al. [15] also described a significantly higher risk for death from heart failure (among other end-points) associated with the Glu27 allele in homozygosis in a similar population than ours, patients with dilated cardiomyopathy. Others, however, have observed exactly the opposite association [16,17]. In particular, Heckbert et al, studying a large sample of elderly individuals without heart failure observed the Glu27 allele to be associated with a lower risk of incident coronary events [18]. Since the Gln27Glu polymorphism may modulate the response to beta-blocker treatment in humans [19], different frequency of beta-blocker use in these different populations and different studied clinical scenarios may explain, at least in part, such discordant findings.

Our study has limitations. Demographic and clinical characteristics of the patients in this study sample may also have been operative for our findings. Specifically, in patients with severe disease in an advanced stage of its natural history, other variables denoting the clinical condition may be more significant for the evaluation of prognosis than a specific genetic substrate. Different etiologies of heart failure in this study sample may also limit the observation. For several baseline predictors we were unable to acquire complete information and we did not have access to follow-up information on medical therapy that could not only modulate overall survival, but also the effect of a particular genotype. Finally, the duration of follow-up was only mid-term.

## Conclusion

The distribution of genotypes of polymorphism Arg16Gly was statistically significant relative to body mass index. We were not able to demonstrate a prognostic influence of polymorphisms Gln27Glu and Arg16Gly of beta-2 receptor gene, as individual alleles or as haplotypes, for the entire spectrum of patients with heart failure. Rather, there appears to be a specific modulating effect of the Gln27Glu polymorphism in individuals older than 65 years and heart failure. Further studies should address the specific role of the Glu27 allele in heart failure deterioration, especially in older individuals.

## Competing interests

The authors declare that they have no competing interests.

## Authors' contributions

AJM conceived the study, participated in the design of the study, data acquisition, interpretation of data and drafted the manuscript; RSF participated in data acquisition; RAC participated in data acquisition; RN conducted initial statistical analysis; APA participated in data acquisition; ACPL participated in data acquisition; JEK participated in the design of the study and supervised molecular analysis; ACP conceived the study, participated in the design of the study, data acquisition, statistical analysis and draft the manuscript. All authors read and approved the final version of the manuscript.

## Pre-publication history

The pre-publication history for this paper can be accessed here:



## Supplementary Material

Additional file 1 **Table S1 and Table S2**. TableS1. Clinical and Demographics characteristics according to genotype Arg16Gly. Tables S2. Clinical and Demographics characteristics according to genotype Gln27Glu. Click here for file
