# Supplementary material for: Beta-2 adrenergic receptor gene polymorphisms Gln27Glu, Arg16Gly in patients with heart failure
Source: BMC Cardiovasc Disord. 2009 Nov 3;9:50. doi: 10.1186/1471-2261-9-50 (PMC2777849; doi:10.1186/1471-2261-9-50)
Supplement: Additional file 1 — Table S1 and Table S2. TableS1. Clinical and Demographics characteristics according to genotype Arg16Gly. Tables S2. Clinical and Demographics characteristics according to genotype Gln27Glu. [file 1471-2261-9-50-S1.doc]

Table S1. Clinical and Demographics characteristics according to genotype Arg16Gly.

|  | Arg/Arg | Arg/Gly | Gly/Gly | P value |
| --- | --- | --- | --- | --- |
| Individuals | 98 | 225 | 179 | - |
| Age, years | 57 (14) | 58 (15) | 58 (14) | 0.77 |
| Gender  Male(%) | 58 | 60 | 60 | 0.94 |
| Ethnicity (%)  Blacks  Mulattos  Whites | 13  12  75 | 13  14  72 | 7  15  77 | 0.75 |
| Etiology (%)  Chagas Disease  Idiopathic  Hypertensive  Isquemic  Heart Failure due to valve disease  Others | 9  10  34  28  14  5 | 14  10  29  25  16  6 | 11  10  25  34  15  7 | 0.78 |
| Biochemical Aspects  Serum Na (mg/dL)  Serum Hemoglobin (mg/dL)  Total Cholesterol (mg/dL)  Triglycerides (mg/dL)  HDL (mg/dL)  LDL (mg/dL)  Creatinin (mg/dL)  Glycemia (mg/dL) | 137 (4)  13 (2)  190 (51)  128 (77)  46 (14)  119 (43)  1,3 (0,5)  115 (56) | 136 (5)  13 (2)  191 (51)  121 (64)  46 (16)  121 (41)  1,4 (1,0)  109 (47) | 137 (5)  13 (2)  189 (52)  123 (65)  44 (14)  120 (43)  1,3 (0,5)  113 (51) | 0,40  0,34  0,94  0,72  0,41  0,93  0,27  0,60 |
| BMI (kg/m²) | 26,3 (5,8) | 25,6 (5,4) | 25,1 (5,1) | 0,34 |
| Heart Rate (pm) | 79 (12) | 79 (13) | 81 (14) | 0,09 |
| Diastolic Blood Pressure (mmHg) | 79 (19) | 73 (18) | 78 (19) | 0,02 |
| Systolic Blood Pressure (mmHg) | 125 (32) | 118 (29) | 125 (33) | 0,04 |

Table S2. Clinical and Demographics characteristics according to genotype Gln27Glu.

|  | Gln/Gln | Gln/Glu | Glu/Glu | P value |
| --- | --- | --- | --- | --- |
| Individuals | 253 | 197 | 48 |  |
| Age, years | 57 (15) | 59 (14) | 59 (15) | 0,41 |
| Gender  Male(%) | 55 | 66 | 60 | 0,08 |
| Ethnicity (%)  Blacks  Mulattos  Whites | 13  15  70 | 9  14  74 | 2  6  92 | 0,14 |
| Etiology (%)  Chagas Disease  Idiopathic  Hypertensive  Isquemic  Heart Failure due to valve disease  Others | 11  11  33  24  15  6 | 13  10  25  32  15  6 | 10  6  19  42  15  8 | 0,38 |
| Biochemical Aspects  Serum Na (mg/dL)  Serum Hemoglobin (mg/dL)  Total Cholesterol (mg/dL)  Triglycerides (mg/dL)  HDL (mg/dL)  LDL (mg/dL)  Creatinin (mg/dL)  Glycemia (mg/dL) | 137 (5)  13 (2)  191 (50)  124 (71)  46 (16)  120 (40)  1,3 (0,5)  112 (54) | 136 (4)  13 (2)  191 (54)  121 (62)  44 (15)  122 (45)  1,4(1)  111 (45) | 137 (5)  13 (2)  188 (50)  129 (63)  44 (12)  116 (40)  1,2 (0,6)  112 (50) | 0,54  0,46  0,92  0,82  0,61  0,70  0,10  0,99 |
| BMI (kg/m²) | 26 (6) | 25 (5) | 26 (5) | 0,18 |
| Heart Rate (pm) | 79 (12) | 80 (14) | 82 (14) | 0,26 |
| Diastolic Blood Pressure (mmHg) | 78 (16) | 74 (19) | 78 (19) | 0,10 |
| Systolic Blood Pressure (mmHg) | 57 (15) | 118 (30) | 122 (31) | 0,08 |
